# Affinity scores: An individual-centric fingerprinting framework for neuropsychiatric disorders

**DOI:** 10.1038/s41398-022-02084-9

**Published:** 2022-08-09

**Authors:** Cassandra M. J. Wannan, Christos Pantelis, Antonia H. Merritt, Bruce Tonge, Warda T. Syeda

**Affiliations:** 1 Melbourne Neuropsychiatry Centre, Department of Psychiatry, The University of Melbourne, Carlton, VIC, Australia; 2 Centre for Developmental Psychiatry and Psychology, Southern Clinical School, Monash University, Clayton, VIC 3168 Australia

**Keywords:** Diagnostic markers, Schizophrenia

## Abstract

Population-centric frameworks of biomarker identification for psychiatric disorders focus primarily on comparing averages between groups and assume that diagnostic groups are (1) mutually-exclusive, and (2) homogeneous. There is a paucity of individual-centric approaches capable of identifying individual-specific ‘*fingerprints*’ across multiple domains. To address this, we propose a novel framework, combining a range of biopsychosocial markers, including brain structure, cognition, and clinical markers, into higher-level ‘*fingerprints*’, capable of capturing intra-illness heterogeneity and inter-illness overlap. A multivariate framework was implemented to identify individualised patterns of brain structure, cognition and clinical markers based on affinity to other participants in the database. First, individual-level affinity scores defined each participant’s “neighbourhood” across each measure based on variable-specific hop sizes. Next, diagnostic verification and classification algorithms were implemented based on multivariate affinity score profiles. To perform affinity-based classification, data were divided into training and test samples, and 5-fold nested cross-validation was performed on the training data. Affinity-based classification was compared to weighted K-nearest neighbours (KNN) classification. The framework was applied to the Australian Schizophrenia Research Bank (ASRB) dataset, which included data from individuals with chronic and treatment resistant schizophrenia and healthy controls. Individualised affinity scores provided a ‘*fingerprint*’ of brain structure, cognition, and clinical markers, which described the affinity of an individual to the representative groups in the dataset. Diagnostic verification capability was moderate to high depending on the choice of multivariate affinity metric. Affinity score-based classification achieved a high degree of accuracy in the training, nested cross-validation and prediction steps, and outperformed KNN classification in the training and test datasets. Affinity scores demonstrate utility in two keys ways: (1) Early and accurate diagnosis of neuropsychiatric disorders, whereby an individual can be grouped within a diagnostic category/ies that best matches their fingerprint, and (2) identification of biopsychosocial factors that most strongly characterise individuals/disorders, and which may be most amenable to intervention.

## Introduction

Population-centric frameworks of biomarker identification focus primarily on comparing averages between groups and assume that diagnostic groups are (1) independent, and (2) homogeneous. However, there is a considerable overlap in symptoms across psychiatric disorders [1–3]. Furthermore, there is now increasing understanding of intra-illness heterogeneity, in which individuals who share a diagnosis can present with a varying array of symptoms and disabilities across a range of clinical, cognitive, structural, and social domains [4, 5]. Intra-illness heterogeneity, combined with overlap of symptoms and frequent comorbidity of psychiatric disorders, can lead to clinically significant delay in receiving the most appropriate treatment, resulting in increased social and functional disability, family burden and accruing societal economic burden [6]. Further, interpretation of neuroimaging-derived biomarkers often requires specialist knowledge, thereby limiting the practical utility of such markers in clinics. Consequently, in recent years, there has been an increased focus on the identification of readily interpretable biomarkers that may characterise specific disorders, and aid in diagnosis, subgrouping, and treatment planning.

Various approaches have been developed to identify hierarchical biomarkers using individual base-level measures, such as regional brain volumes, stress measures and cognitive variables. Such approaches can be broadly classified into three categories: 1) multivariate pattern identification, 2) clustering analyses for machine learning and prediction, and 3) normative models. Although these approaches have contributed strongly towards increasing our understanding of biopsychosocial underpinnings of psychiatric disorders, they offer limited clinical translation. Group-level analyses (e.g., pattern identification, clustering) fail to capture individual complexity spanning over multiple clinical domains, whereas normative approaches are typically performed within a single domain of interest and are based on the assumption that there is a true healthy population to act as a reference group.

Recent approaches have attempted to address some of these limitations by taking a more individual-centric approach to disease identification and classification. One method, developed for use in Alzheimer’s disease, is the Disease State Index (DSI) [7]. The DSI is a statistical modeling and data visualisation system that computes an estimate of an individual’s Alzheimer’s disease state by comparing their biomarker data to previously diagnosed cases. This approach enables individualised multivariate quantification of the disease state; however, an important limitation is that scores are referenced to multiple target populations whilst incorporating information from the entire distributions, and are, therefore, less sensitive to the localised information most closely relevant to the individual-of-interest [8]. One method sensitive to localised information in the data with respect to the participant-of-interest is K-Nearest Neighbours (KNN), a supervised machine learning algorithm [9, 10]. The KNN algorithm assumes that similar data points (e.g., diagnoses) are close to each other, and therefore classifies new data points based on their proximity to *K* nearest neighbours. This approach offers limited reliability in the presence of outliers, as their nearest neighbours could be situated far from them. The weighted KNN algorithm attempts to minimize this effect by applying inverse distance weights to the neighbours. Conversely, in densely populated neighbourhoods, selecting only first *K* neighbours leads to exclusion of information from other nearby neighbours, which can lead to suboptimal performance, especially when there is large information overlap between diagnostic groups.

In order to address the limitations of current approaches, we propose a novel framework capable of capturing intra-illness heterogeneity and inter-illness overlap through combining a range of biopsychosocial markers into higher-level biopsychosocial ‘*fingerprints*’. In this framework, each individual is scored based on their biopsychosocial marker affinity to different diagnostic groups within a fixed sized neighbourhood, thereby generating overlapping indices of group membership. The affinity score framework can incorporate a range of biopsychosocial parameters, including clinical and cognitive measures, measures of global and social functioning, stress and traumatic experiences, genetics, neuroimaging, and other biological and physiological biomarkers, and will allow clinicians to determine which of these factors most strongly contributes to an individual patient’s diagnosis or subgroup and plan intervention strategies accordingly.

In the current study, ‘*affinity scores*’ represent biopsychosocial patterns of treatment-responsive and treatment-resistant schizophrenia, whereby we capture each individual’s affinity to multiple diagnostic groups across a wide range of variables. This individual-level approach has translational value as it allows clinicians to acknowledge comorbidity, indicate severity, disability and prognosis, and tailor intervention strategies to each person’s clinical and biopsychosocial ‘*fingerprint*’. Additionally, we integrated our affinity score framework into two distinct diagnostic verification and classification/prediction algorithms, which can be used as a clinical decision support system designed to enable clinicians to establish a diagnosis and determine a likely prognosis. Our framework was also be compared with an existing framework, K-Nearest Neighbours (KNN) classification, in order to determine the prediction accuracy of our affinity-based classification. Finally, k-means clustering was applied to multivariate Affinity Score profiles in order to assess the separability of treatment-resistant individuals from healthy controls and individuals with chronic schizophrenia.

## Methods

### Participants

Data from 186 individuals with chronic schizophrenia (age: $37.99 \pm 09.98$, 69.9% males) and 168 healthy controls (age: $39.74 \pm 13.97$, 48.2% males) were obtained from the Australian Schizophrenia Research Banks (ASRB) after institutional approval. ASRB is a register of research data collected between 2007 and 2011 by scientific collaborators across five Australian sites [11]. For the purpose of the current study, participants receiving treatment with clozapine (*n* = 37, age: $36.62 \pm 09.86$, 81.1% males) were designated as having treatment-resistant schizophrenia (TRS) [12–14]. ASRB exclusion criteria for participants included: i) a history of organic brain disorder; ii) electroconvulsive therapy in the previous 6 months; iii) current substance dependence; iv) movement disorders; or v) brain injury with post-traumatic amnesia. Healthy controls with a personal or family history of psychosis or bipolar I disorder were also excluded. Detailed information regarding the consent procedures have been published previously [11]. The Diagnostic Interview for Psychosis (DIP) [15] was used to obtain clinical symptom ratings and confirm diagnoses according to ICD-10 or DSM-IV criteria. Study procedures were approved by the Melbourne Health Human Research Ethics Committee and written informed consent was obtained from all participants. Participants with at least one missing variable were excluded from the study, resulting in a total sample size of 182 individuals with chronic schizophrenia (Scz), 136 healthy controls (HCs) and 34 TRS individuals. Demographic data are presented in Table 1.

**Table 1:** Demographics.

	Control		SCZ		TRS				
	Mean	SD	Mean	SD	Mean	SD	Statistic (F)	*p*	Posthoc
Age	39.65	13.84	37.83	9.84	35.82	9.1	1.88	0.154	
IQ	116.74	11.41	105.73	13.68	95.94	15.77	46.43	<0.001	Ctrl > SCZ > TRS
Premorbid IQ	107.03	11.09	99.40	14.05	98.18	17.50	14.41	<0.001	Ctrl > SCZ; Ctrl > TRS
GAF	84.43	9.41	53.78	12.43	50.53	13.28	310.23	<0.001	Ctrl > SCZ; Ctrl > TRS
Positive symptoms			7.43	3.50	7.62	2.58	0.08	0.780	
Negative symptoms			25.33	17.24	30.38	20.16	2.30	0.131	
Illness duration			14.23	9.20	14.18	9.22	0.001	0.977	
	*N*	%	*N*	%	N	%	Statistic (χ^2^)	*p*	
Gender (male)	81	48.2	130	69.9	30	81.1	24.08	<0.001	Ctrl < SCZ; Ctrl < TRS

### Clinical and cognitive measures

The Schizotypal Personality Questionnaire (SPQ) [16] was used to measure schizotypal symptoms in all participants. The Global Assessment of Functioning (GAF) [17] was administered to assess current functioning. Current IQ was measured using the Vocabulary and Matrix Reasoning subtests of the Wechsler Abbreviated Scale of Intelligence (WASI) [18]. Premorbid IQ was measured using the Wechsler Test of Adult Reading (WTAR) [19]. Additional cognitive tasks included Letter Number Sequencing (LNS) [20], the Controlled Oral Word Association Test (COWAT) [21], and five subscales (immediate memory, delayed memory, construction, language, attention) from the Repeatable Battery for Assessment of Neuropsychological Status (RBANS) [22].

### MRI acquisition and processing

T1-weighted (MPRAGE) structural scans were acquired using Siemens Avanto 1.5 T scanners located at five different sites in Australia. The same acquisition sequence was used at each site and a Siemens phantom was periodically imaged at each site to evaluate potential inter-site differences. T1-weighted images comprised 176 sagittal slices of 1 mm thickness without gap; field of view = 250 × 250 mm^2^; repetition time = 1980 ms, echo time = 4.3 ms; data matrix size = 256 × 256; voxel dimensions = 1.0 × 1.0 × 1.0 mm^3^.

To estimate cortical thickness, volume, and surface area, images were processed with the FreeSurfer software package version 5.1 (https://surfer.nmr.mgh.harvard.edu) [23]. In brief, preprocessing included intensity normalization, removal of non-brain tissue, transformation to Talairach-like space, segmentation of gray-white matter tissue, and tessellation and smoothing of the white matter boundary. White matter surfaces were then deformed toward the gray matter boundary at each vertex. The entire cortex of each study participant was visually inspected, and inaccuracies in segmentation were manually edited. The cortical surface was then parcellated into 68 regions based on the Desikan-Killiany atlas [24]. Subcortical volumes (thalamus, amygdala, and hippocampus) and estimated total intracranial volume (ICV) were also obtained using the Freesurfer ‘aseg’ parcellation. A list of regions included in the affinity score model is displayed in Supplementary Table 1.

### Variable-wise affinity scores

The variable-wise affinity scores are calculated on the combined sample from all diagnostic groups. Each variable is converted into z-scores using mean and standard deviation from the pooled data. The variable-specific affinity scores are calculated using the following algorithm.

1. A variable-specific hop-size, $h_v$, is determined in the standard deviation units as,
  1 $$h_v = \frac{{\mathop {\sum}\nolimits_{g = 1}^G {\left| {\mu _{g - 1} - \mu _g} \right|} }}{{\alpha G}},$$
  where, $\mu _g$ is the mean z-score of group *g*, $g = 1,...,G$, and *G* is the number of diagnostic groups in the study. *α* is a scaling constant.
2. For each participant, *p*, the distance from every other participant in the dataset is calculated in the standard deviation units, and a neighbourhood, $\Psi _p^v$, of size $2h_v$ is defined centered at the z-score of the participant $p$, ($z_p \pm h_v$). Participants within the neighbourhood $\Psi _p^v$ contribute towards the group-wise affinity score, $F_{p,g}^v,$ calculated as,
  2 $$F_{p,g}^v = \frac{{f_g}}{{s_g}} \ast \frac{1}{{N_s}}\mathop {\sum}\limits_{g = 1}^G {f_g} ,$$
  where, $f_g$ is the number of participants from group *g* within $\Psi _p^v,g = 1,...,G,s_g$ is the group sample size and $N_s$ is the total number of participants in the data, $N_s = \mathop {\sum}\nolimits_{g = 1}^G {s_g}$.
  For each participant *p* and each variable $v$, the affinity score $F_{p,g}^v$ is a *g* dimensional vector and the magnitude of the *g*th element in $F_{p,g}^v$ describes the strength of affinity to group *g* in the data. The affinity scores can be used for diagnostic verification (participant *p* is included in $\Psi _p^v$) or classification (participant *p* is excluded from $\Psi _p^v$).
3. A variable-specific adjacency matrix, ${{{\boldsymbol{A}}}}^v$, is defined as:

3 $$\begin{array}{l}A_{i,j}^v = 1,j \in \Psi _i^v,j \,\ne\, i\\ \quad \;\; = \,0,{{{\mathrm{otherwise}}}}.\end{array}$$

${{{\boldsymbol{A}}}}^v$ is a matrix of size $N_s \times N_s$ and $i,j = 1,...,N_s.$

### Multivariate affinity

Multiple metrics can be calculated to assess the multivariate distribution of a participant’s affinity to each diagnostic group in the data.

### Composite multivariate affinity

For each participant, a group-specific composite affinity score is calculated by averaging the affinity scores across all variables,

4 $$F_{p,g}^{CA} = \frac{1}{G}\mathop {\sum}\limits_{v = 1}^V {F_{p,g}^v} .$$

The composite affinity scores quantify the average affinity of *p*th participant to each diagnostic group, *g*, in the data. *G* is the number of diagnostic groups in the data and *V* is the total number of variables.

### Rank-based multivariate affinity

For each variable and participant, affinity scores can be ranked based on the affinity strength to each diagnostic group, such that the group with least affinity score is ranked the lowest. The group-wise rank-based average affinity score is calculated as:

5 $$F_{p,g}^{RA} = \frac{1}{G}\mathop {\sum}\limits_{v = 1}^V {r_{p,g}^v} ,$$

$r_{p,g}^v$ is the variable-wise group rank for *p*th participant and takes values in the range $1 \le r_{p,g}^v \le G.$

### Vote-based multivariate affinity

The vote-based affinity score for the *p*th participant is based on a voting system such that each variable votes ($w_p^v$) towards the diagnostic group with maximum affinity:

6 $$w_p^v = max\left( {r_{p,g}^v} \right).$$

The multivariate vote-based affinity score is calculated using the vote-count, such that the diagnostic group with the most votes scores the highest.

### Common neighbourhood-based affinity

The multivariate common neighbourhood is defined as the number of variables in which a pair of participants share a common neighbourhood. The multivariate common neighbourhood adjacency matrix of size $N_s \times N_s$ is defined as

7 $${{{\boldsymbol{A}}}}^C = \mathop {\sum}\limits_{v = 1}^V {{{{\boldsymbol{A}}}}^v} .$$

The group-wise common neighbourhood-based affinity for the $p$th participant is calculated as:

8 $$F_{p,g}^C = \frac{1}{{s_g}}\mathop {\sum}\limits_{k = 1}^{s_g} {A_{p,k}^C} ,$$

where $$g = 1,...,G.$$

### Common community-based affinity

The adjacency matrix of each variable ${{{\boldsymbol{A}}}}^v$ can be used to identify within-variable communities using a clustering algorithm, such as graph clustering with modularity maximization described in (Blondel et al.,) [25]. Each participant is assigned a community, and a participant cannot be a member of multiple communities. The participants within the community share maximum affinity to each other, and each community shares least affinity to other communities. The common community-based affinity scores are calculated by the following algorithm:

1. Within variable communities are identified in the adjacency matrix ${{{\boldsymbol{A}}}}^v$ using a graph clustering algorithm, such as (Blondel et al. 2008) [25], to construct the matrix ${{{\boldsymbol{C}}}}^v$ of size $N_s \times N_s$, such that the subjects within a community are assigned the same label.
  9 $$\begin{array}{l}C_{i,j}^v = 1,i,j \in c_l,j \,\ne\, i\\ \quad \;\; = \,0,{{{\mathrm{otherwise}}}}.\end{array}$$
  $i,j = 1,...,N_s$ and $l = 1,...,N_c^v$, $N_c^v$ is the number of communities within variable $v.$
2. The multivariate common community is defined as the number of variables in which a pair of participants share a common community. The multivariate common community adjacency matrix of size $N_s \times N_s$is defined as
  10 $${{{\boldsymbol{A}}}}^{CC} = \mathop {\sum}\limits_{v = 1}^V {{{{\boldsymbol{C}}}}^v} .$$
3. The group-wise common community-based affinity for the *p*th participant is calculated as:

11 $$F_{p,g}^{CC} = \frac{1}{{s_g}}\mathop {\sum}\limits_{k = 1}^{s_g} {A_{p,k}^{CC}} ,$$

where $$g = 1,...,G.$$

### Implementation on the ASRB data

Data from a total of 195 variables, adjusted for age and sex through generalized linear regression, was used to calculate affinity scores, covering clinical, cognitive, psychosocial and brain structural domains, including cortical and subcortical structures. A complete list of variables and their description is presented in Table 2. For each participant *p* and each variable $v$, the affinity score $F_{p,g}^v$ was a three dimensional vector, with axes corresponding to HC, Scz and TRS groups.

**Table 2:** Clinical and cognitive assessments.

Domain	Test	Variable Description
Cognition	WMS	LNS (Working memory)
	RBANS	Attention
		Constructional
		Delayed memory
		Immediate memory
		Language
		Total
		Total (Scaled)
	WASI	Matrix reasoning
		Vocabulary
	WTAR	Premorbid IQ
	COWAT	Verbal fluency
Clinical	GAF	Global assessment of functioning
	SPQ	Cognitive perceptual
		Interpersonal
		Disorganized
	Demographics	Social functioning
		Social task

*LNS* Letter Number Sequencing, *WMS* Wechsler Memory Scale, *RBANS* Repeatable Battery for Assessment of Neuropsychological Status, *WASI* Wechsler Abbreviated Scale of Intelligence, *WTAR* Wechsler Test of Adult Reading, *COWAT* Controlled Oral Word Association Test, *GAF* Global Assessment of Functioning, *SPQ* Schizotypal Personality Questionnaire.

### Diagnostic verification

The diagnostic verification algorithm was applied to the whole dataset to calculate variable-wise affinity scores using Eqs. 1–2, with each participant included in its neighbourhood. HCs, Scz and TRS individuals were assigned a unique group label. Two diagnostic verification frameworks were developed: a two-group framework (HCs and Scz) and a three-group framework using all three groups. Multivariate metrics of diagnostic accuracy were calculated as described in Eqs. 3–10 to assign affinity-based labels to each individual in the dataset. An individual’s diagnosis was considered verified if the original and affinity-based labels were identical, and atypical if the affinity-based label differed from the original label.

### Classification

To perform affinity-based classification, individuals with TRS were relabeled as Scz, leading to binary classification with groups: HC (*n* = 136) and Scz (*n* = 216). The relabeled data was split into training (110 HCs, 170 Scz) and test (26 HCs, 46 Scz) sets using random selection. 5-fold nested cross-validation was performed on the training set, with hyper-parameter tuning performed in the inner loop on the scaling parameter, *α* (Eq. 1), using grid search: $\alpha \in \{ 1,..,10\}$. In each inner iteration, training data were used to calculate affinity scores, and multivariate affinity-based metrics were calculated (composite affinity, common neighbourhood-based and common community-based metrics). In the validation step, each participant’s affinity-based measures were calculated with reference to the training data, and common neighbourhood-based affinity was used to determine classification accuracy: the number of correctly labelled participants in the validation set divided by the total sample size. The outer loop was used to calculate average classification accuracy across folds.

For comparison, weighted K-nearest neighbours (KNN) classification was performed using MATLAB’s *fitcknn* function, and nested cross-validated on the same partitions of the training data. For weighted KNN classification, weighting was performed using the ‘*inverse distance*’ option on the standardized Euclidean distance between a participant and its *k*th neighbour, and hyper-parameter tuning was performed on the number of neighbours, *K*, using grid search: $K \in \{ 5,...,15\}$. A schema of the nested cross-validation algorithm is presented in Supplementary Fig. S1.

The affinity-based and weighted KNN models were tuned again on the entire training dataset to set hyperparameters, instead of selecting the best-tuned model from the nested cross-validation. As both affinity-based and weighted KNN classifiers are sensitive to the local neighbourhood composition, changes in sample-size require re-tuning of hyperparameters to ensure optimal performance. $\alpha = 6$ and $K = 6$ showed highest accuracy on the training set. The tuned models were applied to the independent test set and prediction accuracy of both classifiers was calculated.

### K-means clustering analyses

To assess the classification separability of the TRS participants, the original group labels were re-applied post-hoc to the multivariate affinity scores from the training data. K-means clustering was performed in MATLAB [26], and three separate clusters were identified using multivariate composite affinity, common-neighbourhood and common-community scores as features. The within cluster group composition was assessed to determine the separability of TRS individuals from other groups.

### Sample size requirements

Sample size calculations of the affinity-based classification were performed similar to the method described in Figueroa et al. [27]. The algorithm used for estimating sample size requirements is presented in the Supplementary Material.

## Results

### Demographic characteristics

As seen in Table 1, there were no differences in age between the groups, however the HC group had significantly fewer males than the Scz and TRS groups. Scz and TRS patients also had significantly lower current and premorbid IQ compared to healthy controls, and TRS patients also had significantly lower current IQ compared to the Scz group. Both the Scz and TRS groups had significantly lower global functioning compared to HCs. No differences in positive and negative symptoms or illness duration were observed between the two patient groups.

### Diagnostic verification

In the two-group diagnostic verification framework, the HCs had verified diagnostic status more frequently when compared to the chronic schizophrenia participants (Table 3). The composite multivariate affinity metric was most sensitive to the heterogeneity within the Scz group, with 64.83% of individuals receiving a verified diagnosis. The vote-based affinity metric was least sensitive to within group heterogeneity, leading to ~100% verified diagnoses.

**Table 3:** Performance evaluation of affinity-based diagnostic verification framework across two diagnostic groups.

Metric	Diagnostic verification (%)			
	HC		Scz	
	Verified	Atypical	Verified	Atypical
Composite multivariate affinity	97.05	02.95	64.83	35.17
Common Neighbourhood-based affinity	94.85	05.15	73.07	26.93
Common Community-based affinity	92.64	07.36	74.17	25.83
Vote-based multivariate affinity	100.00	0.00	100.00	0.00

In the three-group diagnostic verification framework, the HCs and TRS participants had the most frequently verified group membership (Table 4). Similar to the two-group framework, the Scz participants were least likely to achieve a verified diagnosis, potentially due to a higher degree of overlap with both HC and TRS groups and imbalanced sample-sizes, except when the vote-based affinity metric was used (99.5% diagnostic verification). The common community-based affinity metric was the strictest metric, with only 39.56% verified Scz participants.

**Table 4:** Performance evaluation of affinity-based diagnostic verification framework across three diagnostic groups.

Metric	Diagnostic verification (%)					
	HC		Scz		TRS	
	Verified	Atypical	Verified	Atypical	Verified	Atypical
Composite multivariate affinity	97.79	02.21	65.93	34.07	97.05	02.95
Common Neighbourhood-based affinity	92.64	07.36	43.40	56.60	67.64	32.36
Common Community-based affinity	89.70	10.30	39.56	60.44	91.17	08.83
Vote-based multivariate affinity	100.00	0.00	99.45	0.55	100.00	0.00

**Fig. 1:**
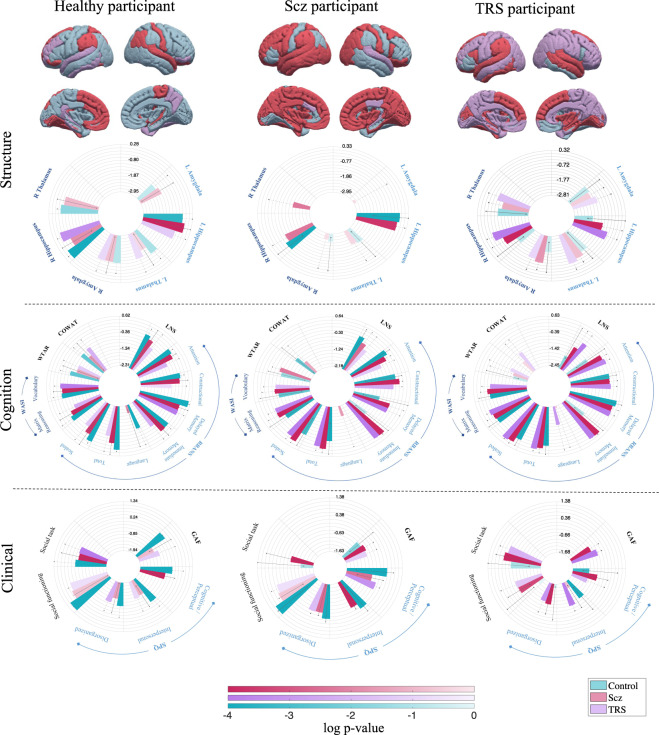
Exemplar individual-centric affinity fingerprints. The cortical pattern describes maximal group affinity within each region. In the circular bar graphs, the variable-wise subcortical, cognitive and clinical affinity scores are plotted on the log-scale with 95% confidence intervals (absent bar: zero affinity score, dotted errorbar: zero lower CI). Shading on the bars is based on the log p-value quantifying score reliability. Within each variable, the largest bar corresponds to the group to which the individual shows strongest affinity, followed by the group with second-highest affinity and so on.

For each individual, the variable-wise affinity scores form a multidomain ‘*fingerprint*’ demonstrating within variable affinity of that individual to each diagnostic group in the data (Fig. 1). The exemplar affinity fingerprint from a healthy participant explains the individualized attributes, with predominantly healthy structural, cognitive and clinical affinities. In contrast, the exemplar fingerprints from chronic schizophrenia and TRS individuals reveal a more heterogeneous pattern of group affinities, providing the evidence for an individual-centric multi-domain multivariate pattern capable of taking intra-illness heterogeneity and inter-illness overlap into account.

### Classification

The affinity scores-based classification achieved a high degree of accuracy in the training, nested cross-validation and prediction steps (Table 5). The common neighbourhood (cross-validated accuracy: 81.79%, prediction accuracy: 84.72%) and common community based (cross-validated accuracy: 79.64%, prediction accuracy: 87.50%) metrics outperformed the weighted KNN algorithm (cross-validated accuracy: 77.86%, prediction accuracy: 77.78%), with the exception of nested cross-validated accuracy in the Scz samples where the weighted KNN classifier achieved slightly higher accuracy (77.65%) than the common-neighbour (75.29%) and common-community (74.12%) affinity-based classification. In the prediction dataset, affinity-based classification achieved higher sensitivity (common-neighbour: 96.15%; common-community: 96.15%) and specificity (common-neighbour: 78.26%; common-community: 82.61%) and more robust positive predictive value (PPV; common-neighbour: 71.43; common-community: 75.76) and negative predictive value (NPV; common-neighbour: 97.30; common-community: 97.44), when accounted for group prevalence in the prediction sample (Table 6).

**Table 5:** Classification accuracy of affinity-based and weighted KNN classifiers in the training, nested cross-validated and prediction datasets. Common neighbourhood and community-based classification outperform composite multivariate affinity and weighted KNN classification.

Classifier	Metric	Accuracy (%)								
		Training			Nested Cross-validation			Prediction		
		HC	Scz	Total	HC	Scz	Total	HC	Scz	Total
Affinity-based Classifier	Composite multivariate affinity	95.45	58.82	73.21	90.91	70.59	78.57	100.00	60.87	75.00
	Common Neighbourhood-based affinity	94.55	71.76	80.71	91.82	75.29	81.79	96.15	78.26	84.72
	Common Community-based affinity	95.45	71.18	80.71	88.18	74.12	79.64	96.15	82.61	87.50
K-Nearest Neighbours	Standardized Euclidean distance	100.00	100.00	100.00	82.73	77.65	77.86	80.77	76.09	77.78

**Table 6:** Performance evaluation of affinity-based and weighted KNN classification in the prediction dataset.

Classifier	Metric	Prediction				
		Accuracy	Sensitivity	Specificity	PPV*	NPV*
Affinity-based Classifier (*α* = 6)	Composite multivariate affinity	75.00	100.00	60.87	59.09	100.00
	Common Neighbourhood-based affinity	84.72	96.15	78.26	71.43	97.30
	Common Community-based affinity	87.50	96.15	82.61	75.76	97.44
K-Nearest Neighbours (*K* = 6)	Standardized Euclidean distance	77.78	80.77	76.09	65.62	87.50

*Adjusted for prevalence in the predict set.

### Separability of TRS participants

**Fig. 2:**
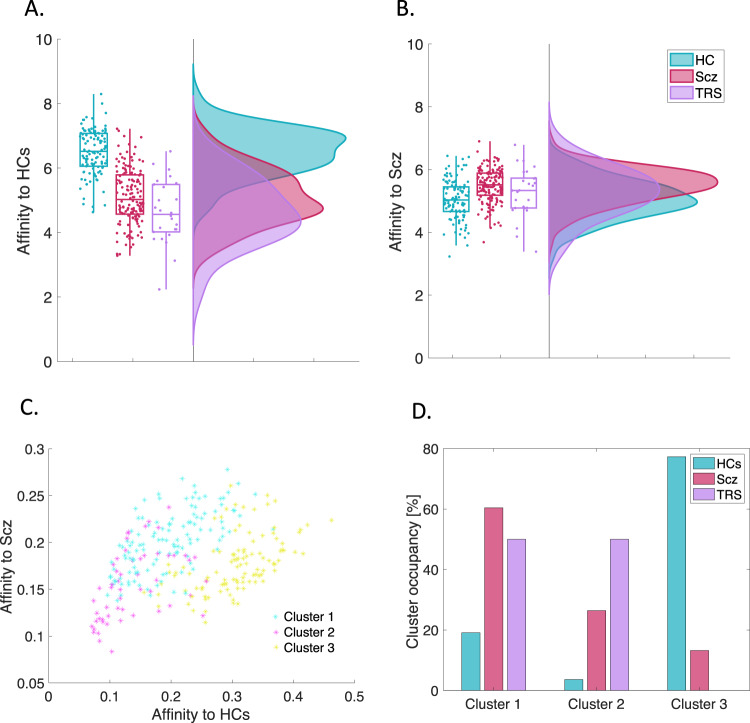
Classification separability of TRS participants from healthy controls and Scz participants in the training dataset (metric: common neighbourhood-based affinity). TRS individuals showed low affinity to both (**A**) HCs and **B** Scz groups. **C** Post-hoc K-means clustering on the multivariate scores from the training dataset used for classification. Three clusters were identified based on affinity to HC and Scz groups using scores from composite affinity, common neighbourhood and common community-based metrics. **D** Group-wise composition of the three clusters (number of participants per group divided by group sample size in the training data).

In the classification step, the separability of TRS participants from the healthy controls and chronic schizophrenia participants was assessed post-hoc by relabeling the TRS participants from Scz to TRS in the training data (Fig. 2). On average, TRS participants showed low affinity to both HCs and Scz participants (Fig. 2A, B), suggesting that TRS participants are separable from these groups. To establish the degree of separability of the TRS participants, k-means clustering was performed on the training dataset using multivariate composite affinity, common-neighbourhood and common-community scores. Three clusters were identified, with cluster 1 predominantly occupied by Scz participants, cluster 2 by TRS participants and cluster 3 by HCs (Fig. 2C, D).

### Sample size requirements

**Fig. 3:**
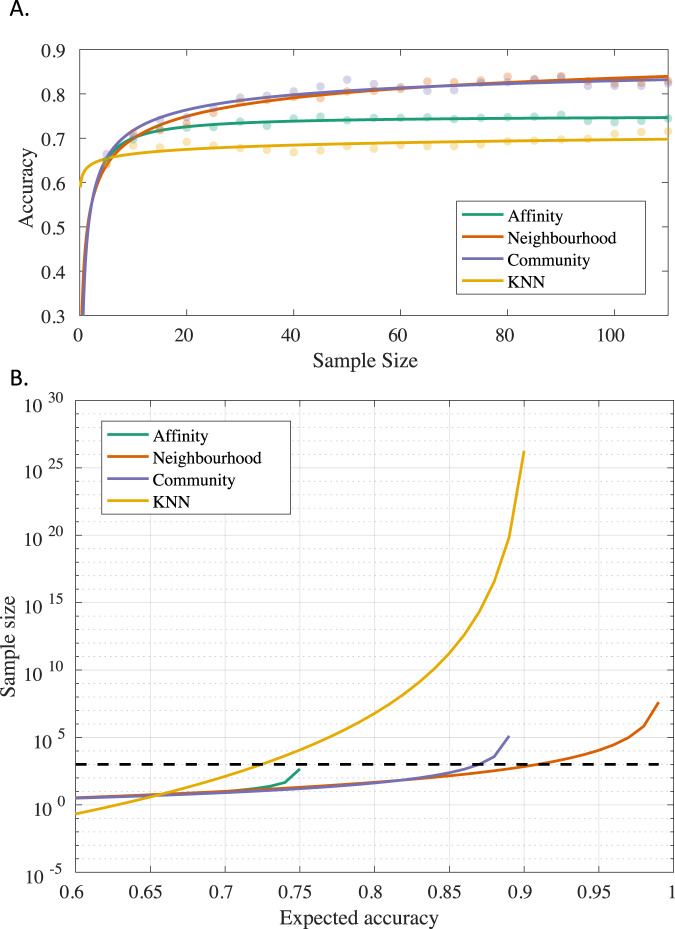
Learning curves for the affinity scores-based classifiers. **A** Learning curves for composite multivariate affinity (green), common neighbourhood-based affinity (red), common community-based affinity (purple) metrics and the weighted KNN classifier (yellow). **B** Sample size as a function of expected accuracy. The common neighbourhood- and community-based metrics achieve higher accuracy compared to the composite multivariate affinity and weighted KNN classifiers. The dotted line is placed at sample size = 1000. For the current study, the common neighbourhood-based classifier outperforms all other classifiers and is expected to reach > 90% accuracy for approx. 1000 participants/group.

The learning curves were estimated for the affinity score classifiers (compositie multivariate affinity, common neighbourhood-based affinity, common community-based affinity metrics) and the KNN classifier (Fig. 3A). The common neighbourhood- and common community-based classifiers achieved higher accuracy values, compared to the composite multivariate affinity and KNN classifiers. The parameter estimates for all learning curves are shown in Table 7. The common neighbourhood-based classifier had the highest maximum achievable accuracy (99%), compared to the KNN algorithm (91%), common community-based (89%) and composite multivariate affinity (75%) metrices. To reach 90% expected accuracy, the common neighbourhood-based classifier had the smallest sample size requirements (692 samples/group) compared to the KNN classifier (1.82e26 samples/group). The sample size requirements over a range of expected accuracies are shown in Fig. 3B.

**Table 7:** Learning curve parameters and sample size estimates of affinity-based and weighted KNN classification.

Classifier	Metric	Learning curve parameters			Max. achievable accuracy, (1-a)	Sample size at 90% accuracy	Sample size at max. achievable accuracy
		Min. achievable error, a	Learning rate, b	Decay rate, c			
Affinity-based Classifier	Composite multivariate affinity	0.25 (0.24, 0.26)	0.51 (0.27, 0.74)	−1.00 (−1.29, −0.71)	0.75	-	462
	Common Neighbourhood-based affinity	5.02e-3 (−0.15, 0.16)	0.55 (0.47, 0.62)	−0.27 (−0.44, −0.09)	0.99	692	4.14e7
	Common Community-based affinity	0.11 (0.038, 0.18)	0.48 (0.37, 0.59)	−0.44 (−0.70, −0.18)	0.89	-	1.30e5
K-Nearest Neighbours	Standardized Euclidean distance	0.09 (−3.59, 3.78)	0.28 (−3.33, 3.89)	−0.06 (−1.06, 0.93)	0.91	1.82e26	3.26e41

## Discussion and conclusion

In this study, we introduce the affinity score framework and examine its utility for accurate verification and classification of schizophrenia-spectrum disorders. An individual’s affinity profile provides a snapshot of their biopsychosocial marker affinity to different diagnostic groups across a wide range of measures, including clinical, cognitive, social, and neuroimaging domains. Furthermore, the classification accuracy of both our verification and classification algorithms was high. Specifically, our verification algorithm was successfully able to verify ~100% of study participants using the voting-based affinity metric, and our classification algorithm successfully classified 91% of healthy controls and 72% of schizophrenia participants.

In two-group analyses, our diagnostic verification approach, which incorporates cognitive, clinical, functioning, and neuroimaging measures, showed very high capability for the verification of schizophrenia and healthy control groupings using the voting-based metric (100% for both groups). However, other metrics, including common neighbourhood- and common community-based affinity were more sensitive to within group heterogeneity, particularly for individuals with schizophrenia, resulting in lower verification capability (73.07% and 74.17% respectively). Similar findings were observed for the three-group analysis, which included TRS individuals. Here, the vote-based metric again showed very high diagnostic verification capability across all three groups. As with the two-group analysis, verification capability was lowest for individuals with schizophrenia, particularly for common neighbourhood- and common community-based metrics, suggesting a greater degree of heterogeneity among individuals with schizophrenia compared to healthy controls or individuals with TRS. Together, these findings suggest that the composite affinity, common neighbourhood- and common community-based frameworks might be useful for identifying intra-diagnosis heterogeneity within the schizophrenia subgroup, where a proportion of patients more closely resemble healthy individuals when the strength of their affinity scores are taken into account. The vote-based metric, on the other hand, while less sensitive to intra-diagnosis heterogeneity, may be particularly useful for diagnostic verification. Together, these metrics would serve as particularly useful clinical decision support tools as they would allow clinicians to determine the overlap between their proposed diagnosis for an individual and the likely diagnosis or diagnoses determined by that individual’s affinity scores. In cases where there is a discrepancy between the clinician’s diagnosis and the diagnosis provided by the verification algorithm, the clinician would be able to determine whether the individual is an atypical presentation of their proposed diagnosis, or whether there may be comorbidities or a differential diagnosis relevant to the individual.

Using all the available biopsychosocial measures, the accuracy of our classification algorithm was also very high. Due to the sample size requirements of classification algorithms, the small number of TRS individuals included in this study were merged with the larger schizophrenia group for the purposes of classification. Unlike the verification algorithm, in which individuals are considered part of their own neighbourhoods, classification requires that individuals are not counted towards their affinity scores, and they are therefore not included in their neighbour counts. In our test sample of 66 schizophrenia participants and 26 healthy controls, our overall classification accuracy was 87.5% for common community-based affinity and 84.72% for common neighborhood-based affinity, which can be compared to 77.78% for the KNN algorithm. The high level of accuracy observed using our classification algorithm suggests this framework has potential as a diagnostic prediction tool in a clinical setting. Of note, when TRS individuals were re-labeled as TRS following affinity score calculation, they did not have high affinity to either the healthy control group or the schizophrenia group. Thus, our algorithm demonstrates the capability of identifying disease subgroups or under-represented populations.

Due to a high degree of conceptual and algorithmic overlap, we compared the performance of affinity-based classification with the weighted KNN classification algorithm. Both algorithms are sensitive to localised information in the data, in contrast to other popular classifiers such as support vector machines, which attempt to identify separation boundaries between classes [28]. Further, prior to classification, no feature selection was performed, and all available variables were included in the diagnostic verification and classification algorithms. Although a more efficient feature selection strategy can potentially lead to improved classification accuracy, less discriminant features still contain clinically-relevant information and excluding such features contradicts the aim of developing a clinically-focused diagnostic support system based on the affinity scores.

In a final step, k-means clustering was applied to multivariate affinity score profiles in order to assess the separability of treatment-resistant individuals from healthy controls and individuals with chronic schizophrenia. The three clusters derived from individual biopsychosocial Affinity profiles included a largely healthy cluster, and two clusters dominated by the schizophrenia and TRS groups. The first of these clusters appears to represent moderate illness severity: the largest contribution to this cluster comes from individuals with schizophrenia, although there is also a relatively large contribution from the TRS group. Without adequate symptom measures it is difficult to fully characterise this group, however, it is possible that the TRS individuals in this cluster are those who have responded to clozapine treatment and are thus functioning at a higher level than those who do not respond to clozapine. The final cluster is largely made up of TRS individuals, with a smaller group of individuals with schizophrenia also present. This cluster most likely represents the most severe illness phenotype, and the individuals with schizophrenia within this cluster may be missed TRS individuals due to the use of clozapine prescription as our grouping measure, or simply a more severely affected subgroup of individuals with treatment-responsive schizophrenia. Thus, as with our verification and classification frameworks, this clustering analysis highlights that, while healthy individuals tend to cluster together, heterogeneity in biopsychosocial profiles exists across the schizophrenia-spectrum.

The affinity score framework has several benefits over more traditional population-based approaches. One important benefit is that this framework is entirely data-driven and does not require individual scores to fall into any particular type of distribution - there is no assumption of “normality”. Furthermore, unlike traditional statistical frameworks, outliers will not have a negative impact on the affinity score algorithm and are considered to be data points of interest rather than scores to be removed or adjusted. Affinity scores can also be dynamic, both at the individual and the population level. At the individual level, affinity scores and ‘*fingerprints*’ can change over time as an individual’s illness evolves, and could potentially be used to predict illness-related outcomes over time. At the population level, the affinity score framework is capable of dealing with the addition of new diagnostic groups and new individuals within existing diagnostic groups, providing a flexible approach to biomarker identification. Thus, although we have developed the affinity score framework in a sample of individuals with schizophrenia, it could be equally applied to other clinical populations, including adolescents and adults, and would have particular utility in the early stages of psychiatric illness where symptoms are often less specific. There is no limit to the number of diagnoses that could be included in a model. This system therefore represents a significant breakthrough in knowledge and practice, substantially improving diagnosis and treatment of psychiatric disorders across diagnostic boundaries.

### Clinical utility of the affinity score framework

Traditional mean-centric approaches are based on assumptions of mutual exclusivity between diagnoses and intra-illness homogeneity and are therefore incapable of taking into account individual-level variation across biopsychosocial measures. Our affinity score framework, on the other hand, takes an individual-centric approach to understanding psychiatric disorders, and is therefore capable of accounting for factors such as comorbidity, and intra-and inter-group heterogeneity and overlap. Across a range of measures, each individual is considered to be the centre of their own unique neighbourhood, with no assumption that the composition of these neighbourhoods will be the same across measures. Thus, one individual may have high affinity to individuals with TRS on one measure, and high affinity to healthy individuals on another. Such an approach allows clinicians to identify not only the most salient treatment targets for any given individual, but also the areas of strength that might be leveraged to enhance treatment efficacy. This approach also allows clinicians to visualise at a glance the diagnostic group/s to which an individual is most closely aligned across multiple variables, aiding in the verification or confirmation of diagnoses and potential comorbidities.

When we examine the ‘fingerprint’ of the Scz patient displayed in Fig. 1, for example, we observe that their working memory and attention scores fall within a neighbourhood predominated by healthy controls, whereas other cognitive functions, such as immediate and delayed episodic memory and verbal fluency, are more closely aligned with Scz and TRS patients. Based on this profile, a clinician may be able to implement treatment strategies that focus on the use of adaptive strategies to work around impairments in memory and verbal fluency. This patient’s social profile also shows some disconnect between their interpersonal functioning, which is strongly aligned with HCs, and their ability to complete daily tasks, such as paying bills, attending appointments, and grocery shopping, which is very strongly aligned with other Scz patients. Thus, a clinician managing this patient may implement an intervention strategy that leverages the patient’s interpersonal relationships to ensure they are carrying out essential tasks, thus improving their global functioning. The TRS patient’s ‘fingerprint’, on the other hand, showed stronger affinity to other TRS and Scz patients across most of the examined measures, with delayed memory the only non-neuroimaging measure to be aligned most strongly with HCs. Thus, for this patient, alongside traditional treatment approaches for TRS, a clinician might implement a psychological and ecological program that encourages delayed learning and capitalizes on this to improve daily living tasks and social engagement.

### Limitations and conclusions

Our findings should be considered in the context of several limitations. In this sample, TRS was defined based on clozapine prescription, however it is possible that some individuals with schizophrenia who are actually treatment-resistant may not have been prescribed clozapine, and therefore would have incorrectly been included in the schizophrenia group. Furthermore, given the small sample size of the TRS group, it was not possible to include these individuals as a separate diagnostic group for the purpose of diagnostic classification and they were therefore merged with the schizophrenia group for this step. However, given that affinity scores are calculated in standardised space, it will be possible to merge in new datasets for future testing and development of the algorithm. In addition, recent developments in harmonization techniques for neuroimaging data, such as the ComBat pipeline [29, 30], will allow for the inclusion of MRI data acquired on different scanners. It is also possible to construct partial fingerprints using only the available measures for a single individual, ensuring missing data does not preclude someone from being included in the Affinity Framework.

The historical categorical, diagnostic approach contrasts with the hierarchical taxometric dimensional method in the description of psychopathology and mental illness [31]. Both systems, however, are based on the grouping of individual symptoms and characteristics, constraining them alternatively to a defined disorder, or to a statistical pattern of characteristics, hierarchically shared by all. Both approaches consign the individual to a collective and fail to adequately capture the individual nature of either their comorbidity and heterogeneity or their specific biopsychosocial aetiology, phenomenology, treatment specificity, and developmental pathway. Individual affinity “fingerprinting” aims to bring an individual focus that bridges the gap between to the categorical and dimensional approaches to understanding psychopathology by defining an individual’s co-morbidities and profile of specific biopsychosocial influences and thus facilitate the tailoring of treatment and inform research and clinical practice. Our novel approach provides a framework on which to develop a clinical support tool to aid clinicians in diagnostic verification and prediction by incorporating multimodal biopsychosocial data. Importantly, unlike traditional prediction algorithms, Affinity Scores not only predict diagnosis and prognosis, but also provide unique, individual-level fingerprints across multiple measures that can be used by clinicians to determine factors that contribute most strongly to an individual’s diagnosis, identify patient strengths and areas of concern, and plan intervention strategies.

## Supplementary information

Supplementary Materials

## References

[1] Ronald A, Simonoff E, Kuntsi J, Asherson P, Plomin R (2008). Evidence for overlapping genetic influences on autistic and ADHD behaviours in a community twin sample. J Child Psychol Psychiatry.

[2] Grisanzio KA, Goldstein-Piekarski AN, Wang MY, Rashed Ahmed AP, Samara Z, Williams LM (2018). Transdiagnostic symptom clusters and associations with brain, behavior, and daily function in mood, anxiety, and trauma disorders. JAMA psychiatry.

[3] Doherty JL, Owen MJ (2014). Genomic insights into the overlap between psychiatric disorders: Implications for research and clinical practice. Genome Med.

[4] Feczko E, Miranda-Dominguez O, Marr M, Graham AM, Nigg JT, Fair DA (2019). The Heterogeneity problem: Approaches to identify psychiatric subtypes. Trends Cogn Sci.

[5] Allsopp K, Read J, Corcoran R, Kinderman P (2019). Heterogeneity in psychiatric diagnostic classification. Psychiatry Res.

[6] Drake RJ, Husain N, Marshall M, Lewis SW, Tomenson B, Chaudhry IB (2020). Effect of delaying treatment of first-episode psychosis on symptoms and social outcomes: a longitudinal analysis and modelling study. Lancet Psychiatry.

[7] Mattila J, Koikkalainen J, Virkki A, Simonsen A, Van Gils M, Waldemar G (2011). A disease state fingerprint for evaluation of Alzheimer's disease. J Alzheimer’s Dis.

[8] Tolonen A, Rhodius-Meester HFMM, Bruun M, Koikkalainen J, Barkhof F, Lemstra AW (2018). Data-driven differential diagnosis of dementia using multiclass disease state index classifier. Front Aging Neurosci.

[9] Ni KS, Nguyen TQ (2009). An adaptable k-nearest neighbors algorithm for MMSE image interpolation. IEEE Trans Image Process.

[10] Triguero I, García-Gil D, Maillo J, Luengo J, García S, Herrera F (2019). Transforming big data into smart data: An insight on the use of the k-nearest neighbors algorithm to obtain quality data. WIREs Data Min Knowl Disco.

[11] Loughland C, Draganic D, McCabe K, Richards J, Nasir A, Allen J (2010). The Australian Schizophrenia Research Bank (ASRB): Quality assurance and control for a comprehensive clinical, neuropsychological, genetic and neuroimaging database for researchers. Aust N Z J Psychiatry.

[12] Suzuki T, Remington G, Mulsant BH, Uchida H, Rajji TK, Graff-Guerrero A (2012). Defining treatment-resistant schizophrenia and response to antipsychotics: A review and recommendation. Psychiatry Res.

[13] Kane J, Honigfeld G, Singer J, Meltzer H (1988). Clozapine for the treatment-resistant schizophrenic. A double-blind comparison with chlorpromazine. Arch Gen Psychiatry.

[14] Howes OD, McCutcheon R, Agid O, De Bartolomeis A, Van Beveren NJM, Birnbaum ML (2017). Treatment-resistant Schizophrenia: Treatment Response and Resistance in Psychosis (TRRIP) Working group consensus guidelines on diagnosis and terminology. Am J Psychiatry.

[15] Castle DJ, Jablensky A, McGrath JJ, Carr V, Morgan V, Waterreus A (2005). The diagnostic interview for psychoses (DIP): Development, reliability and applications. Psychol Med.

[16] Raine A, Wong KK-Y, Liu J. The Schizotypal Personality Questionnaire for Children (SPQ-C): Factor Structure, Child Abuse, and Family History of Schizotypy. Schizophr Bull. 2020. 10.1093/schbul/sbaa100. 10.1093/schbul/sbaa100 PMC8370046 32674122

[17] Jones SH, Thornicroft G, Coffey M, Dunn G (1995). A brief mental health outcome scale-reliability and validity of the Global Assessment of Functioning (GAF). Br J Psychiatry.

[18] Wechsler D. Abbreviated Scale of Intelligence (WASI). Psychological Corporation: San Antonio, TX, 2003.

[19] Wechsler D (2001). Wechsler Test of Adult Reading (WTAR).

[20] Wechsler D. Wechsler Memory Scale - Revised. Psychological Corporation: New York, NY, 1987.

[21] Patterson J. Controlled Oral Word Association Test BT - Encyclopedia of Clinical Neuropsychology. In: Kreutzer JS, DeLuca J, Caplan B (eds). Springer New York: New York, NY, 2011, pp 703–6.

[22] Randolph C, Tierney MC, Mohr E, Chase TN (1998). The Repeatable Battery for the Assessment of Neuropsychological Status (RBANS): Preliminary clinical validity. J Clin Exp Neuropsychol.

[23] Fischl B, Van Der Kouwe A, Destrieux C, Halgren E, Ségonne F, Salat DH (2004). Automatically parcellating the human cerebral cortex. Cereb Cortex.

[24] Desikan RS, Ségonne F, Fischl B, Quinn BT, Dickerson BC, Blacker D (2006). An automated labeling system for subdividing the human cerebral cortex on MRI scans into gyral based regions of interest. Neuroimage.

[25] Blondel VD, Guillaume J-L, Lambiotte R, Lefebvre E (2008). Fast unfolding of communities in large networks. J Stat Mech Theory Exp.

[26] MathWorks I. MATLAB: the language of technical computing: computation, visualization, programming: installation guide for UNIX version 5. Natwick: Math Works Inc., 1996., 1996 https://search.library.wisc.edu/catalog/9910122586102121.

[27] Figueroa RL, Zeng-Treitler Q, Kandula S, Ngo LH (2012). Predicting sample size required for classification performance. BMC Med Inf Decis Mak.

[28] Pisner DA, Schnyer DM. Support vector machine. 2020.

[29] Fortin J-P, Cullen N, Sheline YI, Taylor WD, Aselcioglu I, Cook PA (2018). Harmonization of cortical thickness measurements across scanners and sites. Neuroimage.

[30] Fortin J-P, Parker D, Tunç B, Watanabe T, Elliott MA, Ruparel K (2017). Harmonization of multi-site diffusion tensor imaging data. Neuroimage.

[31] Kotov R, Krueger RF, Watson D (2018). A paradigm shift in psychiatric classification: the Hierarchical Taxonomy Of Psychopathology (HiTOP). World Psychiatry.

