# Supplementary material for: Affinity scores: An individual-centric fingerprinting framework for neuropsychiatric disorders
Source: Transl Psychiatry. 2022 Aug 9;12:322. doi: 10.1038/s41398-022-02084-9 (PMC9363458; doi:10.1038/s41398-022-02084-9)
Supplement: Supplementary file 1 — Supplementary Materials [file 41398_2022_2084_MOESM1_ESM.docx]

**Supplementary Material**

**Methods**

*Sample-size requirements of affinity-based classification*

Sample size calculations of the affinity-based classification were performed using the method described in [(Figueroa et al. 2012)](https://paperpile.com/c/KluWVI/jbNZ). A learning curve was estimated for the affinity based binary classification, as a set of points $\left( x_{j},y_{j} \right), j=1,\ldots,m$, where $m$ is the total number of trials, $x_{j}$ is the training sample size of the $j$th trial, such that $x_{1}<x_{2}\ldots<x_{m}$ The classification accuracy in the $j$th trial , $y_{j}$, can be evaluated using multivariate affinity metrics, such as composite multivariate affinity, common neighbour- and common community-based affinities. The learning curve is described by an inverse power law function:

$y\left( x \right)=\left( 1-a \right)-bx^{c}$, S1

where, $y$ is the classification accuracy using a multivariate affinity-based metric and $x$ is the training sample size. The parameter $a$ describes the minimum achievable error, $b$ is the learning rate and $c$ is the decay rate. The term $1-a$, therefore, describes the maximum achievable accuracy for a given dataset.

For the binary classification using HC and Scz participants, three separate learning curves corresponding to composite multivariate affinity, common neighbour- and common community-based affinities were calculated using the relabeled data split into training (110 HCs, 170 Scz) and test (26 HCs, 46 Scz) sets (same splits were used for classification). A nested iterative algorithm was implemented to control for sequential bias:

In the outer loop (10 iterations), the position of each participant in the training set was randomly permuted, and the permuted training data was employed in the inner loop to estimate a separate learning curve for each iteration.

In the inner loop, training data was sequentially sampled to estimate the learning curve, starting from 5 participants/group and increasing up to 110 participants/group, in 5 participants/group increments. In each iteration, classification accuracy on the independent test set was calculated using the multivariate affinity metrics.

For each multivariate affinity metric, a consensus learning curve was estimated by averaging across training data permutations. The inverse power law function in Eq. S1 was fitted to the consensus learning curve using a nonlinear least squares (trust-region algorithm) solver in MATLAB. The solver was initialised at $a=0.5$, $b=0.8$, $c=0.9$, under the constraints $0\leq a\leq1$, $-inf\leq b\leq inf$ and $-1\leq c\leq0$.

*Permutation testing*

To assess the interchangeability of variable-wise affinity scores between groups, permutation testing was performed with the null hypothesis that affinity scores are interchangeable between groups. An iterative algorithm was implemented:

1. For each iteration, the group labels of all subjects were randomly permuted using MATLAB's *randperm* function.
2. Variable-wise affinity scores were calculated, as described in the Methods section.
3. Steps 1-2 were repeated 10,000 times, with each iteration having randomly permuted group labels.
4. A p-value for the $p$th subject, $g$th group and $v$th variable was calculated based on the total number of times the permuted affinity score was greater than or equal to the estimated affinity score:

$$p_{p,g,v}=\frac{1}{N_{perms}+1}(\sum_{r=1}^{N_{perms}} (|F_{p,g}^{v}|\geq|F_{p,g_{r}}^{v}|) +1),$$

where $g$is the original group label and $g_{r}$is the permuted group label.

*Bootstrapping*

Bootstrapping was performed to estimate confidence intervals of the variable-wise affinity scores, using an iterative algorithm:

1. In each iteration, random sampling with replacement was performed to generate samples from the original data from all variables. The number of samples per group was the same as the original data.
2. For each bootstrap sample, the variable-wise affinity scores were calculated, and the process was repeated 10,000 times to generate a sampling distribution of the variable-wise affinity scores.
3. The 95% confidence intervals were calculated from the sampling distribution.

*Nested Cross-validation*


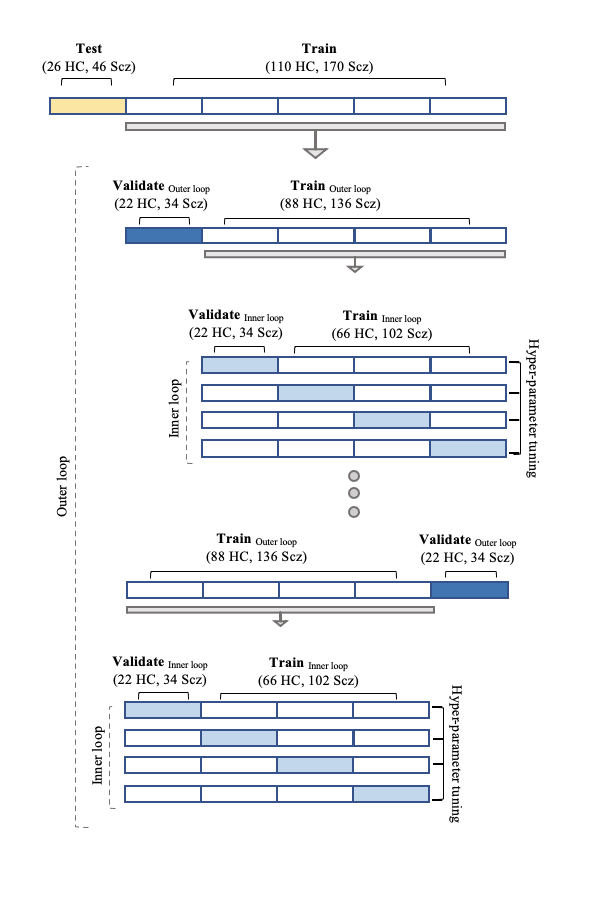


**Figure S1**: Nested cross-validation. 5-fold nested cross-validation was performed on the training set, with hyper-parameter tuning performed in the inner loop on the scaling parameters, $\alpha$and K for the affinity-based and K-nearest neighbours algorithms, respectively. The outer loop was used to calculate average classification accuracy across folds.

**Table S1:** Brain regions included in affinity score model

| Lobe | Region |
| --- | --- |
| Frontal | Superior frontal gyrus |
|  | Rostral middle frontal gyrus |
|  | Caudal middle frontal gyrus |
|  | Pars opercularis |
|  | Pars triangularis |
|  | Pars orbitalis |
|  | Lateral orbitofrontal cortex |
|  | Medial orbitofrontal cortex |
|  | Frontal Pole |
|  | Precentral gyrus |
|  | Paracentral lobule |
| Temporal | Entorhinal cortex |
|  | Parahippocampal gyrus |
|  | Temporal pole |
|  | Fusiform gyrus |
|  | Superior temporal gyrus |
|  | Middle temporal gyrus |
|  | Inferior temporal gyrus |
|  | Transverse temporal cortex |
|  | Banks of the superior temporal sulcus |
| Parietal | Postcentral gyrus |
|  | Supramarginal gyrus |
|  | Superior parietal cortex |
|  | Inferior parietal cortex |
|  | Precuneus |
| Occipital | Lingual gyrus |
|  | Pericalcarine |
|  | Cuneus |
|  | Lateral occipital cortex |
| Cingulate | Rostral anterior cingulate |
|  | Caudal anterior cingulate |
|  | Posterior cingulate |
|  | Isthmus cingulate |
| Subcortical | Hippocampus |
|  | Amygdala |
|  | Thalamus |

**References**

[Figueroa, Rosa L., Qing Zeng-Treitler, Sasikiran Kandula, and Long H. Ngo. 2012. “Predicting Sample Size Required for Classification Performance.” *BMC Medical Informatics and Decision Making* 12 (February): 8.](http://paperpile.com/b/KluWVI/jbNZ)
